# The multifaceted roles of cathepsins in immune and inflammatory responses: implications for cancer therapy, autoimmune diseases, and infectious diseases

**DOI:** 10.1186/s40364-024-00711-9

**Published:** 2024-12-31

**Authors:** Kexin Zhao, Yangqing Sun, Shangwei Zhong, Jun-Li Luo

**Affiliations:** 1 The Cancer Research Institute and the Second Affiliated Hospital, Hengyang Medical School, University of South China (USC), Hengyang, Hunan 421001 China; 2 MOE Key Lab of Rare Pediatric Diseases, Hengyang Medical School, USC, Hengyang, Hunan 421001 China; 3 Department of Oncology, Hunan Provincial People’s Hospital, Changsha, Hunan 410005 China; 4 National Health Commission Key Laboratory of Birth Defect Research and Prevention, Hunan Provincial Maternal and Child Health Care Hospital, USC, Hengyang, Hunan 410008 China; 5 Hunan Provincial Key Laboratory of Basic and Clinical Pharmacological Research of Gastrointestinal Cancer, USC, Hengyang, Hunan 421001 China

**Keywords:** Cathepsins in immune cells, Cancer therapy, Autoimmune diseases, Infectious diseases

## Abstract

The cathepsin family comprises lysosomal proteases that play essential roles in various physiological processes, including protein degradation, antigen presentation, apoptosis, and tissue remodeling. Dysregulation of cathepsin activity has been linked to a variety of pathological conditions, such as cancer, autoimmune diseases, and neurodegenerative disorders. Understanding the functions of cathepsins is crucial for gaining insights into their roles in both health and disease, as well as for developing targeted therapeutic approaches. Emerging research underscores the significant involvement of cathepsins in immune cells, particularly T cells, macrophages, dendritic cells, and neutrophils, as well as their contribution to immune-related diseases. In this review, we systematically examine the impact of cathepsins on the immune system and their mechanistic roles in cancer, infectious diseases, autoimmune and neurodegenerative disorders, with the goal of identifying novel therapeutic strategies for these conditions.

## Introduction

Cathepsins are a diverse group of proteases categorized into three main classes based on their catalytic mechanisms and substrate specificities: cysteine, aspartic, and serine proteases. This family includes several subtypes, such as cathepsins B, D, K, L, S, and C, each exhibiting unique expression patterns and biological functions. Structurally, cathepsins possess a catalytic domain with specific active sites containing catalytic residues. These enzymes can exist as monomers or dimers, and their activity is regulated by post-translational modifications as well as interactions with inhibitors or activators [1, 2].

Cathepsins are predominantly localized in lysosomes, where they play vital roles in protein degradation, antigen presentation, apoptosis, and tissue remodeling, often through secretion into the cytoplasm or extracellular matrix. They are involved in breaking down proteins within lysosomes, thereby contributing to cellular homeostasis and the turnover of intracellular components. Certain cathepsins also participate in extracellular matrix (ECM) degradation, facilitating tissue remodeling during development, wound healing, and pathological processes such as cancer metastasis [3–6].

Recent studies have revealed the complex and essential role of cathepsins in regulating immune responses. For example, cathepsin S is crucial for the maturation of MHC class II molecules during antigen presentation, and emerging evidence suggests it also plays an important role in dendritic cell-mediated T cell activation [7, 8]. Moreover, cathepsin B has been implicated in activating the NLRP3 inflammasome, which is integral to innate immune responses [9]. Certain cathepsins are linked to immune responses, participating in antigen processing and presentation, thereby influencing adaptive immune reactions [10] (Table 1). Furthermore, Emerging research highlights the significant role of cathepsins in immune and inflammatory cells and immune-related diseases, underscoring their potential as therapeutic targets [11].

**Table 1:** Classification and functions of the cathepsin family

classification	cathepsin	function	reference
Cysteine cathepsins	CTSB	A component of the autophagy system	[12]
		Activates NLRP3 inflammasome and contributes to restores macrophage digestion dysfunction	[13]
		β-secretase-like activity and helps to produce amyloid-β (Aβ)	[14, 15]
		Mediates CD18 shedding to regulate leukocyte recruitment from angiogenic vessels	[16]
	CTSL	Helps to increase α-secretase activity	[14, 15]
		Degrades light chains, promotes apoptosis, and facilitates TLR9-mediated signaling in macrophages and neutrophils.	[17]
		Affects the levels of ECM components in lymphoid organs, thymus secretion, and the number of peripheral T cells.	[18]
		Mediates the activation of C3 within the cell to drive Th1 activity.	[19, 20]
	CTSS	Regulates the autophagy process through its interaction with LC3 and ECLIN1.	[21]
		Cleaves Interleukin-36 γ (IL-36 γ).	[22]
		Reduces early chronic airway inflammation and mucus obstruction in βenac-tg mice.	[23]
		Promotes loss of lung function induced by cigarette smoke in mice and regulates the pulmonary inflammatory response.	[24, 25]
	CTSC	Activates the TNF-α/p38 pathway, which in turn, leads to the concentration-dependent activation of CTSC expression by TNF-α.	[26, 27]
		Exacerbates the inflammatory response by inducing microglia M1 polarization through the calcium-dependent PKC/p38MAPK/NF-κB pathway.	[28]
	CTSH	Triggers neuronal cell death.	[29]
		Involved in the production of (Met)enkephalin neuropeptide.	[30]
		Aminopeptidase activity.	[31, 32]
		Involved in the processing of hydrophobic surfactant-associated protein C in type II lung cells.	[33]
		Serves as a negative regulator of apoptosis in insulin-secreting cells.	[34, 35]
		Activates GrB by removing the N-terminal dipeptide Gly-Glu.	[36]
	CTSO	Plays a role in macrophage-mediated matrix remodeling.	[37, 38]
		Plays a role in osteoclast-mediated bone resorption.	[37, 38]
	CTSW	Plays a role in NK-92 cell-mediated cytotoxicity.	[39]
		Correlated with the cGAS-STING pathway and DNA damage repair.	[40]
	CTSF	A prognostic biomarker for pancreatic cancer.	[41–43]
		Involved in the development of dermatitis and various cancers.	[44]
		Inhibits corneal wound healing in diabetic patients.	[45]
	CTSK	The major collagen-degrading enzyme in osteoclasts, it dissolves dense collagen fibers and rebuilds the extracellular matrix by degrading MMP-9.	[46–49]
	CTSV	Remodels the ECM by cleaving adhesion molecules such as fibronectin, E-cadherin, and N-cadherin to promote lung cancer metastasis, and inhibits T cell activity in vitro.	[50]
		Promotes the proliferation of bladder cancer through the NF-κB pathway.	[51]
		Inhibits the expression of GATA3, which is essential for normal breast development.	[52, 53]
		Mediates elastolysis by macrophages.	[54]
	CTSX	LFA-1 is the substrate of CTSX activity, which mediates cell-cell interactions and leukocyte adhesion.	[55, 56]
Aspartic Cathepsins	CTSD	CTSD levels were positively correlated with the proinflammatory cytokines IL-8 and TNF-α.	[57]
		Overexpressed in chronic obstructive pulmonary disease (COPD), it cleaves antimicrobial peptides (AMPs) through its proteolytic activity.	[58]
		Involved in the activation and cleavage of vascular endothelial growth factor C (VEGF-C).	[59]
	CTSE	A key downstream effector of TGR5 in regulating inflammatory immune responses.	[60]
		Complex regulatory roles within immune cells.	[61]
Serine cathepsins	CTSA	Protects the functionality of neuraminidase-1 (NEU1).	[62]
		A therapeutic target for preventing left ventricular remodeling.	[63]
	CTSG	Pro- and anti-inflammatory activities, including regulation, bactericidal, and destructive functions.	[64, 65]
		Involved in the antigen presentation of proinsulin.	[66]
		Cleaves the surface glycoproteins of red blood cells and directly inhibits the invasion of parasites.	[67, 68]
		Inhibits colon cancer progression through negative regulation of the Akt/mTOR/Bcl2 pathway.	[69]

Despite these insights, a comprehensive understanding of how cathepsins regulate immune cell functions remains incomplete. In this review, we aim to systematically examine the regulatory roles of cathepsins in immune cells, focusing on their functions in T cells, macrophages, dendritic cells, myeloid-derived suppressor cells (MDSCs), and neutrophils. We will discuss how cathepsins influence immune cell activation, differentiation, and function, and explore the underlying mechanisms. By providing an integrated overview, we seek to clarify the complex interactions between cathepsins and immune cells, underscoring their significance in immune regulation.

Additionally, we will explore how dysregulation of cathepsin activity in immune cells contributes to the pathogenesis of immune-related diseases, such as cancer, infectious diseases, autoimmune and neurodegenerative disorders. By understanding these mechanisms, we hope to identify potential therapeutic targets within the cathepsin family that could be leveraged for treating these conditions.

## The roles of cathepsins in immune cells

### The roles of cathepsins in T cells

#### The roles of cathepsins in T cells activation and antigen presentation

CD8 + T cells recognize cancer cells through the TCR/CD3/Major Histocompatibility Complex I (MHC-I) pathway, while CD4 + T cells rely on MHC-II molecules. The T Cell Receptor (TCR) recognizes antigens presented by MHC-I on the surface of dendritic cells (DCs) or cancer cell membranes, transmitting signals to the associated CD3 complex, which then plays a role in intracellular signaling [70]. It has been observed that the aggregation of MHC-I on the surface of cancer cells decreases during tumor progression, impairing the antigen-presenting function of MHC-I [71].

Cathepsin S (CTSS) is involved in processing both MHC-I and MHC-II pathways, as well as in cross-presentation by specialized antigen-presenting cells (APCs) [72, 73]. Studies in mice and humans have demonstrated that various non-specific APCs, such as cancer-associated fibroblasts (CAFs), utilize a CTSS-dependent vacuolar pathway to present synthetic long peptides (SLPs) of neoantigens to cytotoxic CD8 + T cells. This process can suppress T cell function by reducing their cytotoxicity and activation while enhancing their consumption [74]. In patients with follicular lymphoma (FL), recurrent hotspot mutations targeting tyrosine 132 (Y132D) in CTSS enhance its activity, affecting antigen processing and disrupting the interaction between T cells and malignant B cells, which contributes to the development of lymphoid tumors [75]. The accumulation of genomic alterations in malignant B cells promotes the interaction and proliferation of tumor-associated immune cells, facilitating immune evasion [76]. Conversely, CTSS deficiency may induce CD8 + T cell activation and increase antigen diversity, thereby enhancing anti-tumor immune responses, particularly in aggressive lymphomas [75].

#### The roles of cathepsins in the formation and function of class II–associated invariant chain peptide (CLIP)

Antigen presentation is vital for immune tolerance and pathogen/tumor recognition. APCs present exogenous peptides to CD4 + T cells via MHC II molecules, initiating immune responses. The processing and maturation of MHC II molecules are crucial for effective antigen presentation and link innate and adaptive immunity [77]. Cathepsin L (CTSL) and CTSS degrade the invariant chain (Ii) in endosomes and lysosomes, forming CLIP, essential for loading antigenic peptides onto MHC II [78, 79]. CTSL targets leucine motifs, while CTSS cleaves the CLIP region [78, 79]. The MHC II-associated invariant chain (Ii) regulates MHC II transport and prevents premature peptide binding. Ii is processed into CLIP, which facilitates antigen presentation on the cell surface [80, 81].

**Fig. 1:**
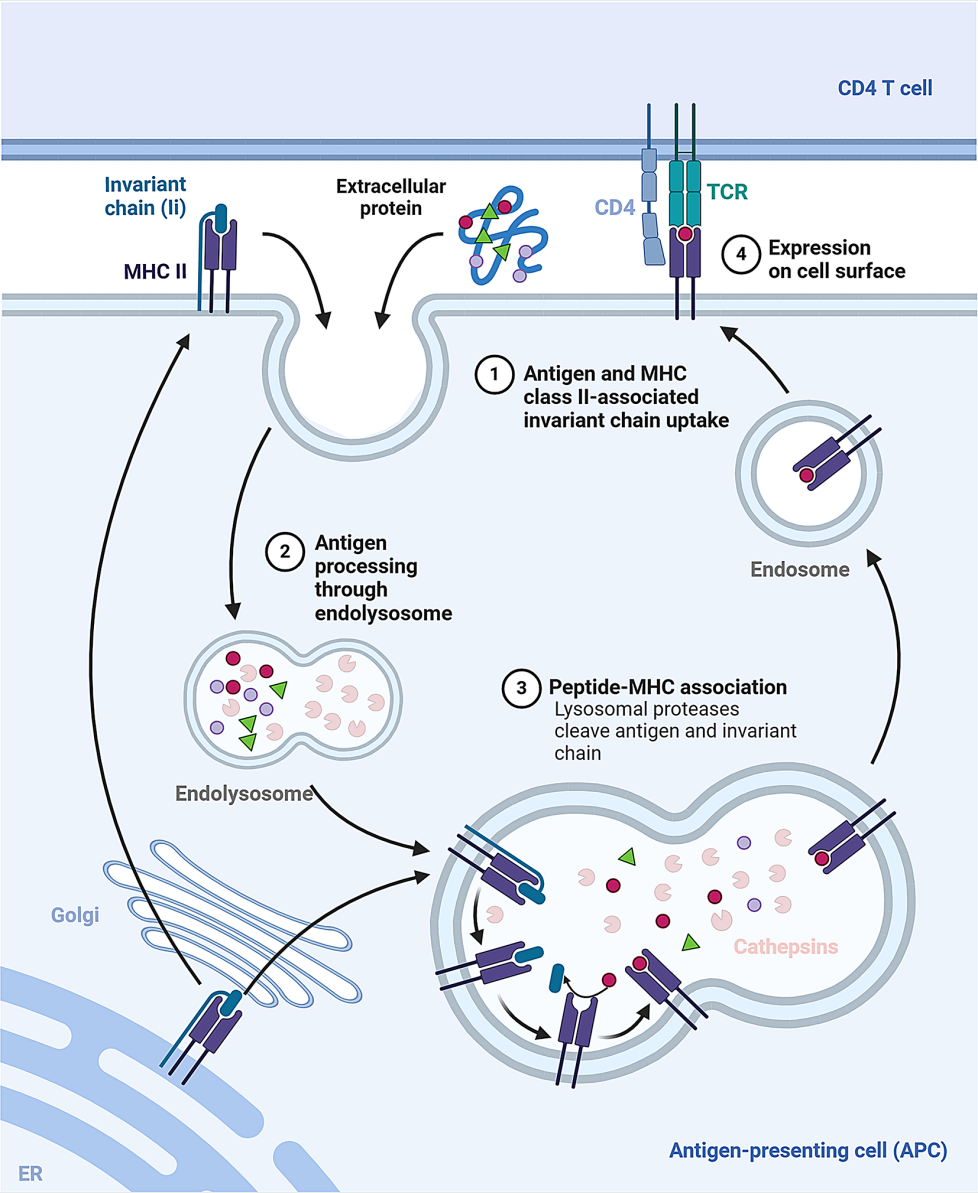
Cathepsins in the processing and maturation of MHC II molecules. During the maturation of MHC II molecules, the invariant chain is progressively degraded, leaving behind a peptide segment known as CLIP. CLIP acts as a “placeholder” within the antigen-binding groove of the MHC II molecule, preventing the binding of non-specific peptides until the MHC II molecule can bind to the appropriate antigenic peptide. This process occurs in the cell’s endoplasmic reticulum and endosomes and involves the fusion of endosomes with lysosomes, where lysosomal cathepsins play a role in degrading the invariant chain

The cathepsins involved differ across APCs: CTSS degrades Ii to CLIP in B cells and dendritic cells, while CTSL, CTSS, and cathepsin F (CTSF) contribute in thymic epithelial cells (TECs) and macrophages. CTSL specifically handles this in the thymus, and CTSS processes it in peripheral lymphoid organs. CTSF is involved in lip10 processing in macrophages [7, 82–85]. CTSS is crucial for antigen presentation in dendritic cells, and cathepsins regulate the transition from lip10 to CLIP. Recent research shows that promoting CTSS-mediated antigen processing with Bacillus Calmette-Guérin (BCG) inhibits melanoma metastasis and extends survival [86].(Fig. 1).

#### The roles of cathepsins in MHC

Cysteine cathepsins, including CTSS, CTSL, and cathepsin B (CTSB), degrade both antigens and B cell receptors (BCRs), playing key roles in generating peptides for Human Leukocyte Antigen (HLA) class II molecules. In the acidic endolysosomal compartments of APCs, these cathepsins break down CNS proteins and immunoglobulin G (IgG) into peptides that can be presented to T cells from the CNS and monoclonal antibodies [87].

Traditional Chinese medicines, such as Cycloastragenol (CAG) from *Astragalus membranaceus*, show anti-tumor effects. CAG targets CTSB, inhibiting its degradation of MHC-I in lysosomes and promoting MHC-I repolymerization on cell membranes. This enhances CD8 + T cell activation via the TCR/CD3/MHC-I pathway, boosting anti-tumor immunity and inhibiting tumor progression [88]. Elevated levels of cathepsin H (CTSH) in colon cancer patients have been linked to MHC class II antigen presentation [89]. The expression of lysosomal thiol-reductase (GILT) in melanoma cells activates cathepsins B, S, and D [90], enhancing antigen processing and stimulating CD4 + T cells. Cathepsins B and S are crucial for processing peptides presented by HLA class II molecules and degrading melanoma antigens [91].

Cathepsins have a dual role in immunity. Under normal conditions, they support immune response by aiding antigen presentation via MHC molecules. However, in the tumor microenvironment, overexpressed cathepsins can facilitate immune evasion by altering antigenic peptide profiles, reducing tumor-specific antigen presentation. Tumor cells may also use cathepsins to degrade T cell surface receptors and co-stimulatory molecules, disrupting T cell-APC interactions. Targeting cathepsins could therefore be a potential strategy for tumor immunotherapy.

Cathepsin V (CTSV) and CTSL are structurally similar, with around 80% homology, but differ in tissue distribution [92]. While CTSL is widely expressed, CTSV is predominantly found in the thymus and testis [93]. CTSV mRNA levels in the thymus are 75 times higher CTSL levels. Like CTSL, CTSV is essential for degrading invariant chains during MHC-II antigen presentation, particularly in the thymic epithelial cells (TECs). Abnormal CTSV overexpression in the thymus linked to myasthenia gravis (MG), a condition involving thymic lesions [94]. This suggests that defects in T cell selection could increase autoreactive cells, potentially contributing to MG development [92].

#### The roles of cathepsins in Treg cells

Regulatory T cells (Tregs) are a subset of T cells with immunosuppressive functions that help maintain immune balance. Notably, Tregs treated with CTSS inhibitors promoted tumor apoptosis more effectively than treated with PBS, contradicting findings from physiological conditions. One hypothesis posits that in Tregs, CTSS mediates the processing of Toll-like receptor 7 (tLR7), enhancing immunosuppressive activity. However, targeting CTSS in Treg cells in the tumor microenvironment may convert them into immunoreactive cells, boosting the number and activity of immune cells, particularly cytotoxic CD8 + T cells. This reduces Treg apoptosis and enhances CD8 + T cell function in tumor. Thus, while CTSS inhibition in Tregs may suppress immune responses in normal conditions, it seems to boost CD8 + T cell activity in cancer [95]. Recent studies also highlight cathepsin W (CTSW), induced by TGF-β during peripheral Tregs (pTregs) development, as a negative regulator that cleaves the IL-2R subunit CD25, inhibiting pTreg cell function, stability, and generation. CTSW deficiency increases Foxp3 expression during pTreg cell differentiation, enhancing immunosuppression [96]. The regulation of cathepsins is complex, influenced by both their expression and environmental factors. While most research targets cathepsins in cytotoxic T cells, emerging studies suggest their potential in Tregs. Understanding of cathepsin’s role in immune cells is crucial for developing new immunotherapies and preventing autoimmune diseases.

### The roles of cathepsins in macrophages

Macrophages exhibit remarkable plasticity, allowing them to polarize into pro-inflammatory (M1) or anti-inflammatory (M2) phenotypes, which can interconvert based on environmental cues. Tumor-associated macrophages (TAMs) play a key role in tumor progression and immune evasion, often correlating with poor prognosis and chemotherapy resistance [97]. Manipulating cathepsin activity to shift macrophage polarization offers promising therapeutic opportunities. For example [98], inhibiting specific cathepsins can convert macrophages from a tumor-promoting M2 state to an anti-tumor M1 state, enhancing tumor suppression in cancers with high M2 infiltration [99].

The inhibitor GB111-NH2 that targets CTSB, CTS and CTSL can induce a shift from M2 to M1 macrophages, evidenced by changes in lysosomal activity, fatty acid metabolism, and pro-inflammatory mediator synthesis. This strategy counteracts the pro-tumorigenic effects of the tumor microenvironment (TME) and improves patient outcomes [100]. CTSL, produced by both tumor cells and macrophages, promotes breast cancer metastasis by driving M0 to M2 differentiation via IL-4 [101]. Chemotherapy agents like naphthyl platin reset M2 macrophages to an M1 phenotype, enhancing susceptibility to colorectal cancer by altering CTSL function and autophagy through the MEK/ERK1/2 pathway [102].

Additionally, O-GlcNAc transferase (OGT) in TAMs promotes tumor metastasis and chemoresistance by driving CTSB maturation through glucose uptake and the hexosamine biosynthesis pathway. M2 TAMs, which have high glucose uptake, are particularly susceptible to this process [103]. Cathepsin K (CTSK) mediates interactions between intestinal microbiota imbalance and colon cancer, where its overexpression and the presence of M2 TAMs form a feedback loop that worsens prognosis [104]. CTSK activates macrophages to secrete IL-4, IL-10, and IL-17, promoting tumor growth and invasion [105, 106]. CTSK also activates the mTOR pathway, essential for TLR4-mediated M2 macrophage recruitment [104, 107].

### The roles of cathepsins in dendritic cells

#### The roles of cathepsins in DCs

DCs are divided into two main types: conventional DCs (cDCs) and plasmacytoid DCs (pDCs), with cDCs further categorized into cDC1 and cDC2 subgroups. Each type has distinct phenotypes and functions. pDCs, which differentiate in the bone marrow, are known for producing large amounts of type I interferons and inflammatory cytokines as danger signals [108]. cDC1 cells are particularly effective in activating and polarizing T cells, especially through cross-presention of exogenous antigens via the class I pathway, while pDCs are more proficient at type I interferon production [109, 110]. Mature thymic cDCs excel at processing and presenting foreign antigens to CD4 and CD8 T cells, whereas splenic cDCs are more responsive to danger signals that activate Toll-like receptors (TLRs) [111].

Cathepsins, including CTSS and CTSL, are major antigen-processing enzymes in DCs [112], with their expression being regulated by tissue type and subpopulation specificity [113]. CTSL is nearly undetectable in pre-cDCs and both cDC subpopulations but is highly expressed in bone marrow pDCs. This expression increases in thymic and splenic pDCs, indicating CTSL’s role in pDC development. Additionally, the expression of CTSL in splenic cDCs can be upregulated after TLR agonist stimulation, reaching levels similar to those found in thymic cDCs [113].

#### Comparison of the impact on DC and macrophages-mediated immune responses

While the role of conventional cathepsins in the immune system is becoming clearer, cathepsin E (CTSE) exhibits distinct, and even opposing, effects in DCs versus macrophages. CTSE-deficient mice exhibit impaired immunity, with fewer tumor-infiltrating macrophages compared to controls [114]. This deficiency leads to increased oxidative stress and mitochondrial dysfunction in macrophages, including reduced ATP levels, mitochondrial membrane depolarization, and reduced mitochondrial oxygen consumption [115]. CTSE-deficient macrophages secrete higher levels of soluble lysosomal proteins, including CTSB, and accumulate lysosome-associated membrane proteins (LAMP-1 and LAMP-2), resulting in elevated lysosomal pH [116]. Consequently, their ability to phagocytose and degrade ovalbumin (OVA) and respond chemotactically to MCP-1 and FMLP is impaired. In contrast, CTSE deletion in DCs improves antigen presentation. This enhancement is due to the reduced activity of L-Aspartic Acid protease, bringing it closer to the optimal levels for OVA processing. Although CTSE does not directly process antigens, it regulates the endosomal/lysosomal microenvironment and protein sorting. The increased expression of co-stimulatory molecules such as CD86, CD80, and CD40 in CTSE-deficient DCs enhances T cell activation, highlighting CTSE’s crucial role in DC-mediated immune responses [61, 117].

### The roles of cathepsins in MDSCs

MDSCs are a heterogeneous population of immature myeloid cells that suppress T-cell responses and promote tumor immune evasion [118]. Perforin, a pore-forming protein in natural killer (NK) cells and CTL, is synthesized as an inactive precursor and becomes active when cleaved in acidic lysosome-like cytotoxic granules. This cleavage is pH-dependent, and while CTSL preferentially cleaves near the C-terminus of perforin, it is not the only protease involved [119]. Similarly, cathepsin X (CTSX) cleaves C-terminal peptides, such as trimming the leukocyte-specific β2 integral linker receptor. This modification influences MDSC interactions by adjusting the receptor’s affinity for extracellular ligands [120]. Recent studies show that targeting CTSL activity increases CD8 + T cell toxicity, while inhibiting CTSX restores tumor cell invasion. Interestingly, inhibiting these cathepsins in either MDSC or tumor cells alone did not produce these effects, suggesting a need for deeper investigation into how cathepsins regulate MDSC-tumor cell interactions in cancer therapy [121].

Chemotherapy also triggers MDSCs to secrete IL-1β through lysosomal permeabilization and CTSB release, which in turn induces IL-17 secretion from CD4 + T cells, reducing the chemotherapy’s effectiveness [122]. The neuraminidase inhibitor LCL521 activates CTSB and CTSD, leading to autophagic vesicle accumulation and dysfunctional autophagosomes, a potential new mechanism of MDSC cell death [123]. In the intestinal epithelium, cathepsins also play a crucial role in HLA class II-mediated antigen presentation to CD4 + T lymphocytes, underscoring their importance in immune regulation [124].

### The roles of cathepsins in neutrophils

Neutrophil extracellular traps (NETs), initially recognized for antimicrobial defense, have recently been linked to cancer progression. Tumor-derived factors trigger NET formation through cathepsin-dependent pathways [125]. In hepatocellular carcinoma, NET-derived cathepsin G (CTSG) promotes tumor cell invasion by regulating adhesion molecules and inflammatory mediators, a process that can be disrupted by DNAzyme I therapy, indicating its therapeutic potential [126–128]. CTSG exhibits functional diversity in cancer, activating pro-caspase-7, interactng with gasdermin D (GSDMD) to promote regulated cell death, and enhancing neutrophil migration by converting proMMP-1 to active MMP-1 [129, 130].

CTSG, alongside mast cell chymase, plays a role in pathogens killing and induces apoptosis in cardiomyocytes, affecting fibroblast migration and differentiation. In myocardial IR injury, CTSG and chymase inhibitors reduce inflammation, apoptosis, and fibrosis, improving heart function and reducing heart failure progression [131–135]. In ischemic vascular injury, CTSG cleaves the PAR4 thrombin site, activating PAR4 and blocking pathological platelet-neutrophil interactions [136, 137]. CTSG also mediates the direct tumor killing by neutrophils through the receptor for advanced glycation end products (RAGE), though RAGE-CTSG inhibition still allows partial cytotoxic effects, suggesting other synergistic components [138].

**Fig. 2:**
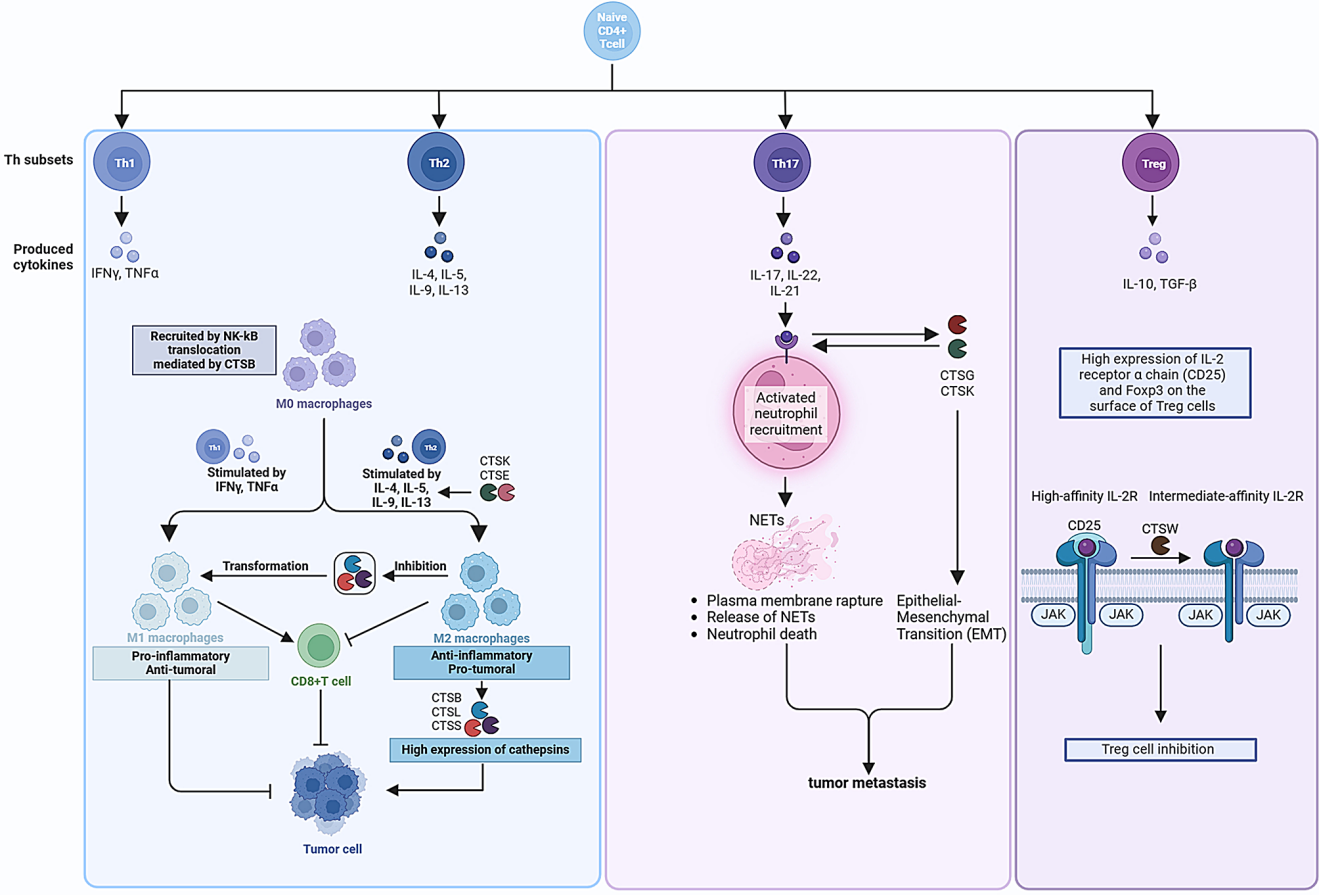
The regulatory role of cathepsins in immune cells influences disease progression. Naive CD4 + T cells, when activated through TCR engagement and co-stimulation, differentiate into Th1, Th2, Th17, and Treg cells, each producing a distinct set of cytokines. Th1 cells produce IFNγ and TNFα, which drive macrophages to differentiate into the M1 phenotype, whereas Th2 cells secrete IL-4, IL-5, IL-9, and IL-13, promoting the M2 macrophage phenotype. Cathepsins modulate this differentiation process; their inhibition can shift M2 macrophages to the M1 phenotype, potentially curbing cancer progression. Th17 cells synthesize IL-17, IL-21, and IL-22, which induce the formation of NETs, a process regulated by cathepsins and associated with tumor metastasis promotion. Treg cells produce IL-10 and TGF-β, but their suppressive function can be compromised by the cleavage of the surface molecule CD25 by CTSW

The CTSC-PR3-IL-1β axis regulates neutrophil recruitment and activation. CTSC processes proteinase 3, activating IL-1β and initiating NF-κB signaling, which amplifies neutrophil recruitment to tumor sites through chemokine production, particularly CCL3 [139]. AZD7986, a selective CTSC inhibitor has shown reduced toxicity and effectiveness in reducing lung metastases in tumor models [140]. These findings suggest that targeting cathepsin-dependent pathways could provide novel cancer therapies (Table 2; Fig. 2).

**Table 2:** Researches for the roles of cathepsins in various immune cells

Immune cells	Function	CTSA	CTSB	CTSC	CTSD	CTSE	CTSF	CTSG	CTSK	CTSL	CTSV	CTSS	CTSW	CTSX
T cell	Antigen cross-presentation [74]		√		√							√		
	Degradation of the MHC class II-associated invariant chain [79–81]		√				√			√	√	√		
	Degradation of MHC-I in lysosomes [88]		√											
	Processing of HLA Class II [87, 91, 124]		√									√		
	Tregs inhibit effector T cells [141]											√	√	
Macrophages	Activate macrophages [106, 107]		√									√		
	Phagocytic function [142]		√	√								√		
	Promote macrophage differentiation [101, 143]							√		√				
	M2 macrophages transform into M1 phenotype [101, 102, 105]		√							√		√		
Dendritic cells	Processing antigen [144–146]									√		√		
Myeloid-derived suppressor cells	Promote tumor development [120, 121, 147]		√		√					√				√
Neutrophils	Neutrophil extracellular traps [139, 148]			√				√						

### The regulatory role of cathepsins in immune-related diseases

#### Cathepsins in tumor

Inflammation is a critical driver of cancer development, often associated with chronic inflammation. The interplay between inflammation and cathepsins represents a fundamental axis in cancer development [149]. For instance, LPS-TLR4 signaling enhances the expression and activity of cathepsin (e.g., CTSB, CTSL, CTSS) through MyD88-dependent and -independent pathways. TLR4 induced cytokines increase proteolytic activity without altering mRNA expression, reflecting changes in the acidic compartments of the cell [150, 151]. In the tumor microenvironment, cathepsins regulate tissue protease functions through inflammatory mediators, CTSB, for example, is associated with immune cell infiltration and immunosuppression, while the interaction of CTSX and γ-enolase affects neuroinflammatory and neurodegenerative diseases [152, 153]. CTSX has emerged as a critical mediator in tumor invasion through its distinctive RGD motif-dependent mechanisms [154]. Secreted by both cancer cells and TAMs, CTSX regulates tumor proliferation and invasion, with macrophage-derived CTSX specifically contributing to the invasive phenotype This functional dichotomy, driven by differential subcellular compartmentalization of the RGD motif, suggests CTSX as a promising therapeutic target to prevent tumor metastasis [155].

In hepatocellular carcinoma (HCC), upregulation of cathepsin A and H correlates with poor prognosis. Elevated levels of CTSH are associated with lung, prostate, colorectal, and breast cancers, influencing cancer progression through apoptosis regulation and signaling pathways such as Apelin and Hedgehog. These molecules can serve as tumor prognostic biomarkers [156–163]. In hematological malignancies, such as follicular lymphoma, CTSE inhibits tumor growth and metastasis by cleaving TRAIL on tumor surfaces. Over 80% of HCC have underlying hepatic fibrosis [164], macrophages are the main source of hepatic CTSS, which acts as a novel necrosis factor, remodeling the extracellular matrix and activating hepatic stellate cells via integrin α5β1 signaling, contributing to hepatic fibrosis. Mutations in CTSS (e.g., Y132) enhance enzyme activity and shift the immune microenvironment toward a pro-tumor state [165–167].

In hormone-dependent cancers, such as breast cancer, CTSD promotes tumor cell proliferation and apoptosis through through various mechanisms, including the regulation of PI3K-mTOR signaling [168, 169], recruitment of immune cells like M2 macrophages, and inhibition of NK cells, all of which remodel the tumor microenvironment to promote tumor growth [170]. Genetic factors, including SNPs in ZNF423 and cathepsin O (CTSO), influence susceptibility to ERα + breast cancer and response to SERM therapy, highlighting the potential for personalized medicine [171–173]. In prostate cancer, CTSK is influenced by the CD200-CD200R axis and the IL-17 A-CTSK-EMT axis, which are involved in tumor progression and metastasis [174, 175]. Overexpression of LRP5 in Wnt signaling can inhibit CTSK expression, offering a potential treatment for bone metastasis in breast cancer [176].

In CNS malignancies like glioma, cathepsins B, D, and S serve as biomarkers to differentiate tumor grades [177, 178]. High CTSA expression correlates with increased M2 macrophage infiltration in tumors, making it a potential biomarker for glioma risk, progression, and prognosis [179]. Recent advances in cathepsin biology have unveiled promising therapeutic opportunities in cancer treatment, including both standalone and combinatory approaches with other immunotherapy [180–182]. Thus, cathepsins have potential as biomarkers to guide treatment decisions.

### Cathepsins in infectious diseases

#### Cathepsins in viral infection

**Fig. 3:**
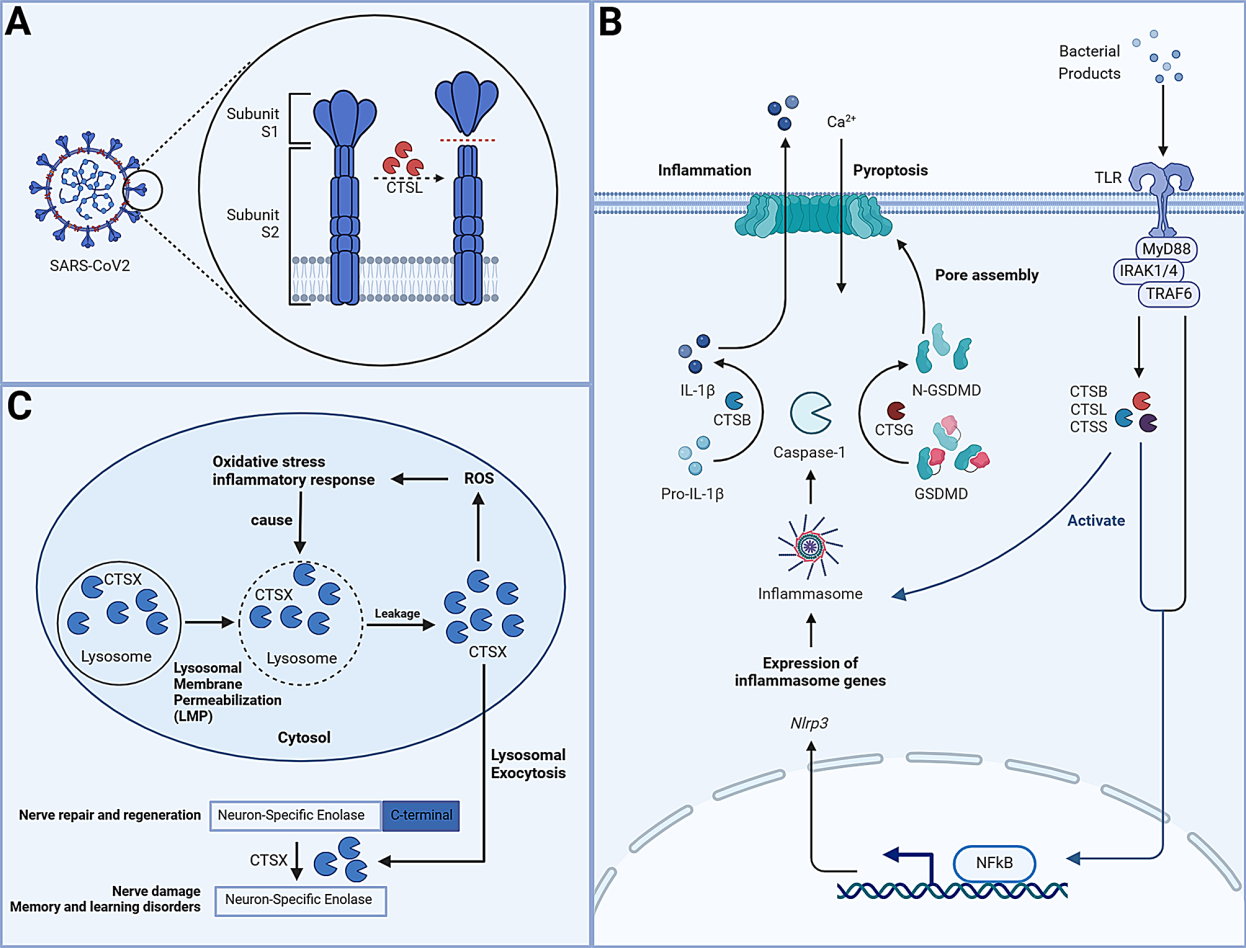
Mechanisms by which cathepsins exert their effects in various diseases through multiple molecular patterns. **(A)** Cathepsin X-induced lysosomal leakage results in the cleavage of the C-terminal domain of neuron-specific enolase (NSE), causing its dysfunction and leading to neuroinflammation and neurodegeneration. This process also enhances the production of reactive oxygen species (ROS), causing oxidative stress and inflammatory responses, which create a positive feedback loop with lysosomal membrane permeabilization (LMP). **(B)** Activation of Toll-like receptors (TLRs) leads to the activation of the transcription factor NF-κB, which promotes the formation of the NLRP3 inflammasome. This facilitates the transcription and translation of pro-inflammatory cytokine precursors such as pro-IL-1β and mediates pyroptosis through GSDMD. **(C)** The cleavage of the S2 subunit of the SARS-CoV-2 spike protein is a critical step for viral entry into host cells, occurring primarily at the S1/S2 cleavage site and mediated mainly by cathepsin L

CTSL plays a critical role in the infection process of SARS-CoV-2 [183]. Elevated CTSL expression is associated with immune dysfunction in SARS-CoV-2-infected patients, correlating with immune cell infiltration (e.g., CD8 + T cells, B cells, CD4 + T cells, neutrophils, macrophages, and dendritic cells) [184]. SARS-CoV-2 infection promotes CTSL expression and activity, which cleaves the viral spike protein, facilitating viral invasion. Circulating CTSL levels may therefore reflect disease progression and severity [185, 186]. The main protease (Mpro) of SARS-CoV-2 is a key target for antiviral therapies, and dual inhibitors that target both Mpro and CTSL show promise in overcoming drug resistance and effectively inhibiting viral replication [187, 188]. SARS-CoV-2 replication requires an acidic environment, optimal for cysteine proteases like CTSL [189] (Fig. 3A). Inhibiting CTSL blocks viral entry and replication [190]. However, long-term CTSL inhibition may lead to tissue fibrosis and impair CD4 + T cells selection in the thymus, highlighting the need for careful clinical optimization [191, 192].

Severe COVID-19 pneumonia, marked by high inflammation, is linked to dysregulated immune responses, including the activation of interferon-stimulated genes, monocyte/macrophage activation-associated genes, the complement pathway, and CTSC [193]. Targeting CTSC may help prevent irreversible lung failure in severe COVID-19 cases [194]. Moreover, HIV can evade T cell recognition by binding to CTSS cleavage sites, resulting in the destruction of immunodominant epitopes and subsequent immune escape [195, 196].

### Cathepsins in bacterial infection

Mycobacterium tuberculosis (Mtb) alters its intracellular environment by modulating lysosomal cathepsins and their inhibitors, such as cystatins [197]. During Mtb infection, CTSB is released from lysosomes and activates the NLRP3 inflammasome, crucial the innate immune response [198]. CTSB both activates NF-κB to produce pro-IL-1β and also hydrolyzes pro-IL-1β via caspase-1, facilitating its maturation [199, 200] (Fig. 3B). Cystatin C, a natural cathepsin inhibitor regulates cathepsin activity by competitively binding to their active sites in phagocytes during infection [197].

Non-pathogenic Mtb strains do not induce the broad cathepsin downregulation seen in pathogenic strains. Research indicates that many cathepsins contribute to Mtb killing [201], while pathogenic strains interfere with macrophage cathepsins. This function can be restored by cystatin F and C, enhancing Mtb killing when cathepsin inhibitors are silenced [202, 203]. The bacterial protein Rv3364c secreted by Mtb-infected macrophages inhibits membrane CTSG, preventing apoptosis [204]. Infected human macrophages generally downregulate cathepsin levels, whereas non-pathogenic M. smegmatis infections upregulate them [201]. CTSG can directly kill bacteria but is downregulated by Mtb, aiding bacterial survival within monocytes [205]. Genetic factors also influence CTSG expression in macrophages infected with mycobacterium avium subspecies paratuberculosis (MAP) [206].

Cathepsins in lysosomes are crucial for antigen presentation and inducing adaptive immune responses [205]. For example, during Helicobacter pylori infection in gastric mucosa, CTSX expression increases, particularly in gastric carcinoma, where it enhances tumor invasiveness by hydrolyzing proteins that regulate cell behavior [207]. Mice lacking CTSX exhibit increased epithelial proliferation and macrophage infiltration, suggesting a complex and potentially protective role for CTSX in inflammatory conditions [208]. In Salmonella Typhimurium (STm) infection, cathepsins are transported to the nucleus, correlating with DNA fragmentation and cell death. Increased gasdermin D (GSDMD) expression during STm infection highlights cathepsin involvement in cell death through atypical inflammasome pathways [209].

### Cathepsins in autoimmune diseases

CTSS plays a dual role in autoimmune diseases, particularly in MHC class II-mediated presentation of autoantigens, which is crucial for activating autoantigen haptens [210]. CTSS, secreted by various immune cells, can also promote endothelial injury through PAR2 signaling, indicating that inhibiting CTSS may slow autoimmune diseases progression [211]. Its role in antigen presentation and potential anti-inflammatory effects may be beneficial for early treatment [212].

In renal allograft rejection, CTSS contributes to alloantigen recognition by activating recipient alloreactive T cell proliferation. While prophylactic CTSS inhibition has been effective in suppressing some inflammation during acute kidney transplant rejection, it does not fully address interstitial inflammation, likely due to its specific involvement with MHC class II rather than MHC class I pathways [79]. Nonetheless, targeting CTSS shows promise in treating autoimmune diseases such as Sjögren’s syndrome, particularly for alleviating dry eye symptoms [213].

In multiple sclerosis (MS), pain management is critical. Studies in experimental autoimmune encephalomyelitis (EAE) models suggest that CTSE plays a key role in neuroimmune communication via neutrophils. CTSE processes elastase, activating pain signaling pathways in dorsal root ganglion neurons. Mice deficient in CTSE show reduced mechanical pain sensitivity, suggesting its importance in pain modulation in MS [214]. In MS patients, the CCR6 chemokine receptor, expressed by activated memory CD4 + T cells, facilitates CNS access. Unique epitope peptides expressing CCR6 are associated with the CDR3 region, which is predicted to be cleaved by CTSS or CTSB [215].

The CTSE gene exhibits higher expression in MRL/lpr lupus-prone mice compared to control C57BL/6 mice. Hypomethylation and mutation in intron 1 disrupt full Kaiso binding and reduce its recruitment. Increased expression of CTSE and IL-10 on CD4 + T cells may contribute to SLE pathogenesis [216]. However, further research is needed. Some studies suggest that defective mitochondria and excessive autophagy in SLE patients increase T cell apoptosis [217]. Overexpression of CTSE may compensate by upregulating defective mitochondria, suppressing autophagy, and mitigating lupus severity [216].

In experimental autoimmune myocarditis (EAM), CTSG binds to Dipeptidyl Peptidase-4 (DPP-4) on T lymphocytes, enhancing its activity by inhibiting SerpinA3N. This activates the Renin–Angiotensin System, releasing proinflammatory cytokines, chemokines, and reactive oxygen species (ROS), with angiotensin II as a major CTSG substrate. Upregulation of angiotensin II by CTSG exacerbates EAM, highlighting its role in disease progression [218].

### Cathepsins in neurodegenerative diseases

Cathepsins play a key role in neurodegenerative diseases by degradating misfolded or aggregated proteins, a hallmark of these conditions [219], alongside enzymes like neuron-specific enolase (NSE), cathepsins are critical in these processes. In Alzheimer’s disease (AD), CTSB exhibits β-secretase-like activity, contributing to amyloid-β (Aβ) production, a key factor in AD pathogenesis. CTSL enhances α-secretase activity, influencing amyloid precursor proteins processing. Both CTSB and CTSL are potential therapeutic targets for AD due to their roles in Aβ production and processing [152, 220].

Cathepsins are crucial in the autophagy-lysosomal pathway, essential for neuronal survival and function. Disruption of this pathway results in the accumulation of dysfunctional organelles and proteins, exacerbating neurodegeneration [169, 221]. In neurodegenerative diseases and traumatic brain injury, lysosomal membrane permeabilization (LMP) can occur, causing CTSB to translocate to the cytoplasm. This may promote apoptosis by generating tBid activate pro-inflammatory cytokines IL-1β and IL-18, contributing to neuroinflammation and neurodegenerative lesions [222, 223].

NSE, an enolase isozyme involved in nerve repair, activates cysteine carboxypeptidase CTSX, which cleaves NSE’s C-terminus, leading to neuroinflammation and degeneration. Inhibiting NSE reduces CTSX levels and neuronal death, suggesting a therapeutic approach for preventing neuroinflammation and promoting nerve regeneration, especially after spinal cord injury [224, 225] (Fig. 3C).

In addition, γ-Enolase and CTSX are co-expressed in basement membrane tissues, primarily associated with macrophages and microglia. CTSX cleaves γ-enolase, counteracting its neurotrophic activity. Microglia, act as CNS macrophages, play a crucial role in immune responses and maintaining CNS homeostasis [226, 227]. These findings highlight the complex roles of cathepsins and related enzymes in neurodegeneration, with potential as therapeutic targets for diseases like Alzheimer’s.

## Cathepsins as therapeutic targets for immune-related diseases

### Small molecule inhibitors of cathepsins

Research increasingly highlights the role of cathepsins in immune response-mediated tumorigenesis, neurodegeneration, inflammatory responses, and autoimmune diseases, making them key targets for therapeutic strategies. Small molecule inhibitors, such as E-64, CA-074Me, ODANACATIB, and Balicatib, are widly used to target cathepsins in treating tumors and other diseases. E-64 irreversibly inhibits CTSB and CTSL [228]. CA-074Me, a derivative of CA-074, inactivates both CTSB and CTSL in mouse fibroblasts, showing therapeutic potential for tumors and viral myocarditis [229]. CA-074 selectively targets CTSB‘s carboxyl peptidase activity, reducing tumor cell metastasis in tumor-bearing mice [230]. ODANACATIB specifically inhibits CTSK, reducing bone resorption, increasing bone mineral density, and decreasing fracture risk, thus aiding in the treatment of osteoporosis [231]. Effective in inhibiting CTSK, Balicatib demonstrates positive outcomes in osteoporosis, with changes in trunk morphology after 9 months of treatment. However, its selectivity decreases over time due to lysosomotropic accumulation, leading to broader inhibition of various cathepsins [232].

### Implications of cathepsins in photodynamic therapy (PDT)

PDT uses photosensitizers for targeted, minimally invasive treatment. These photosensitizers generate ROS upon light excitation, causing cytotoxic effects. Dual-stimuli activated photosensitizers, such as glutathione (GSH)-responsive 2,4-dinitrobenzenesulfonate (DNBS)-substituted zinc (II) phthalocyanine units linked by Gly-Phe-Leu-Gly (GFLG) peptide chains, are cleaved by CTSB. Pre-treatment with a CTSB inhibitor reduces fluorescence intensity of the probes, preventing activation by free phthalocyanine units and facilitating targeted cancer therapy [233]. A multifunctional nano-probe is activated by CTSB is used for targeted treatment of triple-negative breast cancer (TNBC). The GFLG peptide linker, selectively cleaved by TNBC-derived CTSB, releases the photosensitizer SQ for fluorescence imaging and enhanced PDT efficacy. This nano-probe integrates tumor-extracellular matrix (ECM) targeting and endogenous enzyme activation, offering a novel approach for TNBC treatment [234].

Sonodynamic Therapy (SDT) is an emerging modality with potential clinical applications. For prostate cancer treatment, a nano-formulation designed for targeted SDT utilizes CTSB overexpression in the tumor microenvironment to enhance ultrasound-induced cytotoxicity, showing increased toxicity at acidic pH levels [235].

## Conclusions

This review highlights the multifaceted roles of cathepsins in regulating immune cell functions, underlining their significance in both innate and adaptive immunity. In the MHC-II pathway, cathepsins regulate the maturation of MHC-II molecules through cleavage of invariant chains, which in turn influences antigen cross-presentation by APCs [79]. Targeting macrophage cathepsins can shift their phenotype from M2 type, which promotes tumor progression, to M1 type, which inhibits tumor growth, thus impacting tumor invasiveness and immune evasion [99]. Cathepsins also modulate MDSCs in the tumor microenvironment, dampening immune responses [236], and facilitate neutrophil recruitment, NET release, and tumor metastasis [148]. Additionally, cathepsin dysregulation is implicated in autoimmune and infectious diseases, with viruses such as HIV exploiting cathepsins to undermine immune function by disrupting antigen presentation [195, 196].

These findings emphasize cathepsins as crucial regulators of immune cell activity and promising therapeutic targets. Combining cathepsin inhibitors with chemotherapy, radiation, immunotherapy, or other drugs holds the potential to enhance treatment outcomes [237–239]. However, while endogenous inhibitors like cystatins provide some level of regulation [240, 241], their broad inhibition of cathepsin across the family limits their specificity [242]. Designing selective inhibitors remains challenging due to the structural similarities and overlapping substrate specificities among cathepsins. To date, no FDA-approved cathepsin inhibitors are available for cancer treatment, and existing inhibitors have not demonstrated sufficient anticancer effects in clinical settings.

Future studies should focus on better understanding the mechanisms by which cathepsins regulate immune cell functions, identifying specific substrates and pathways, and developing targeted therapies capable of modulating cathepsin activity with high specificity.

## Data Availability

No datasets were generated or analysed during the current study.

## Abbreviations

CTSA: Cathepsin A
CTSB: Cathepsin B
CTSC: Cathepsin C
CTSD: Cathepsin D
CTSE: Cathepsin E
CTSF: Cathepsin F
CTSG: Cathepsin G
CTSH: Cathepsin H
CTSK: Cathepsin K
CTSL: Cathepsin L
CTSO: Cathepsin O
CTSS: Cathepsin S
CTSW: Cathepsin W
CTSX: Cathepsin X
CTSV: Cathepsin V
IL-36γ: Interleukin-36γ
COPD: Chronic obstructive pulmonary disease
AMP: Antimicrobial peptide
VEGF-C: Vascular endothelial growth factor C
NEU1: Neuraminidase-1
MHC-I: Major Histocompatibility Complex I
MHC-II: Major Histocompatibility Complex II
TCR: T cell receptor
APC: Antigen-presenting cell
CAF: Cancer-associated fibroblast
SLP: Synthetic long peptide
Y132D: Tyrosine 132
CLIP: Class II–associated invariant chain peptide
Ii: MHC Class II-associated invariant chain
CAG: Cycloastragenol
GILT: Lysosomal thiol-reductase
TEC: Thymic epithelial cell
MG: Myasthenia gravis
TLR: Toll-like receptor
PREP: Prolyl endopeptidase
ECM: Extracellular matrix
NSP: Neutrophil serine protease
H3ΔN: Histone H3 amino-terminus
TME: Tumor microenvironment
TAM: Tumor-associated macrophage
OGT: O-GlcNAc transferase
HBP: Hexosamine biosynthetic pathway
SCD: Sudden cardiac death
IR: Ischemia and reperfusion
CRC: Colorectal cancer
DC: Dendritic cell
cDC: Conventional DC
pDC: Plasmacytoid DC
BM: Bone marrow
TFH: Follicular helper T
CTL: Cytotoxic T lymphocyte
ECTV: Ectromelia virus
LAMP: Lysosome-associated membrane protein
MDSC: Myeloid-derived suppressor cell
NET: Neutrophil extracellular trap
NF-κB: Nuclear factor kB
CCL3: C-C motif chemokine ligands 3
HCC: Hepatocellular carcinoma
GSDMD: Gasdermin D
RAGE: Receptor for advanced glycation end product
LPS: Lipopolysaccharide
FL: Follicular lymphoma
SARS-CoV: Severe acute respiratory syndrome coronavirus
HNSCC: Head and neck squamous cell carcinoma
Mpro: Main protease
Mtb: Mycobacterium tuberculosis
MAP: Mycobacterium avium subsp paratuberculosi
MDM: Monocyte-derived macrophage
SPEM: Spasmolytic polypeptide-expressing metaplasia
STm: Salmonella typhimurium
PAR: Protease-activated receptor
SS: Sjögren’s syndrome
MS: Multiple sclerosis
EAE: Experimental autoimmune encephalomyelitis
DRG: Dorsal root ganglion
NE: Neutrophil elastase
SLE: Systemic lupus erythematosus
EAM: Experimental autoimmune myocarditis
DPP-4: Dipeptidyl peptidase-4
CNS: Central nervous system
NSE: Neuron-specific enolase
AD: Alzheimer’s Disease
Aβ: Amyloid-β
ROS: Reactive oxygen species
LMP: Lysosomal membrane permeabilization
PDT: Photodynamic Therapy
GSH: Glutathione
GFLG: Gly-Phe-Leu-Gly
TNBC: Triple-negative breast cancer
SDT: Sonodynamic Therapy
